# Oral Presentations 6

**DOI:** 10.1038/sj.bjc.6601934

**Published:** 2004-06-22


### ORAL PRESENTATIONS 6

### 6.1

### A SENSITIVE AND REPRODUCIBLE METHOD FOR IDENTIFICATION OF RADIATION-INDUCED MUTATIONS: IMPLICATIONS FOR RADIATION TREATMENT &AN&PROTECTION

M Boyd , K Hughes , A Livingstone , AM Clark , N Baig , L Wilson , S Ross , K Prise , RJ Mairs
[1] United Kingdom, CRUK Beatson Labs, Glasgow, [2] United Kingdom, Department of Clinical Physics, Glasgow, [3] United Kingdom, Gray Laboratories, London

Characterisation of the mutations induced by ionising radiation is required to improve radiation protection, to identify radiotherapy patients whose normal tissues are abnormally prone to radiation damage and to optimise radiotherapy and limit radiation-induced second tumours. Hypervariable tandemly repeated nucleotide sequences (mini- and microsatellites) have been shown to be mutable by radiation and several studies have indicated an increase in the minisatellite mutation rate in human and murine germ cells post irradiation.
Utilising these sequences, we have developed a sensitive and reproducible methodology, Automated Fluorescent Fragment Analysis, for analysis of radiation mutagenesis in somatic cells. We identified a radiation dose-response relationship for mutation induction in UVW human glioma cells and observed that, with respect to detection of genetic aberrations, the analysis of hypervariable repeat sequences was 100 to 1000 fold more sensitive than conventional methodology utilising coding loci. We detected a 4-fold higher mutation rate in hypervariable loci after treatment of cells with 0.3 Gy rather than 3 Gy gamma radiation. This finding has implications for the initiation of radiation-mediated second tumours as a result of the low dose exposure of normal tissues during radiotherapy.
In order to assess the environmental implications of EMF population exposure, we are currently using our novel methodology for the analysis of mutations induced by EMF radiation. We are also applying this sensitive assay to mutation detection in individuals who are heterozygous for the Ataxia Telangiectasia (AT) gene in order to determine whether low penetrance genes may render such individuals susceptible to radiation mediated cancers. These studies may lead to modification of radiotherapy treatment planning and identify subsets of the population unsuitable for radiation-based medical screening procedures involving X-rays.

### 6.2

### THE MOLECULAR PATHOLOGY OF POST-CHERNOBYL THYROID CANCER: IS THERE A RADIATION SIGNATURE?

GA Thomas
South West Wales Cancer Institute, University of Wales on behalf of CHIPS EC consortium, Swansea, United Kingdom

The incidence of papillary thyroid cancer (PTC) in those areas exposed to fallout from the Chernobyl accident has increased dramatically, particularly in those under 19 at exposure. To determine whether there is a radiation signature, we studied the molecular pathology of post Chernobyl thyroid cancer using a variety of techniques on material supplied by the Chernobyl Tissue Bank (www.chernobyltissuebank.com).
Ret rearrangement was studied by RT-PCR in 100 papillary cancers from patients aged under 15 at the time of the Chernobyl accident; 44% of these harboured ret rearrangements; the frequency in PTCs from children in England and Wales is 48%. There was no correlation with either age at exposure or latency. We also studied 43 of these tumours using FISH for ret. We found that the majority of PTCs harboured positive cells with the translocation, and the maximum number of positive nuclei was 55%. There was a discrepancy between cases positive by RT-PCR (41%) and FISH (72%). Further investigation showed that 31% of cases showed clustering of positive nuclei, suggesting subclonal growth. Sampling of different areas of the tumour is likely to account for differences between FISH and RT-PCR results.
Expression analysis of 2400 genes was also performed 12 cases of PTC using Micromax cDNA array and compared with 9 PTCs from Belgium and France. Hierarchical clustering analysis on the 50% most expressed genes showed no differences between the 2 groups of tumours. Permutation analyses revealed differences in the expression of 10 genes; 5 of these related to lymphoid infiltration in the tumours, the other 5 showed small differences in expression, and are therefore likely to be of no biological significance. These results suggest there is no molecular signature for radiation induced thyroid cancer. The molecular biological features of post Chernobyl PTCs may relate more to the age of the patients studied, rather than their aetiology.

### 6.3

### THE INDUCTION OF TUMOUR HYPOXIA BY VASCULAR DISRUPTING AGENTS AND THE IMPLICATIONS FOR COMBINATION WITH RADIATION THERAPY

M.R. Horsman , R. Murata
Dept. Experimental Clinical Oncology, Aarhus University Hospital, Aarhus, Denmark

**Introduction:** Combining vascular disrupting agents (VDAs) with conventional treatments is a novel approach for cancer therapy. But, the VDA-induced reductions in tumour blood flow could increase hypoxia that may impact the other therapy. This study investigated the induction of hypoxia by VDAs and what affect that had on radiation response.
**Methods:** A C3H mammary carcinoma grown in the right rear foot of female CDF1 mice was used when at 200 $mm^3$. The VDAs, injected i.p., were combretastatin A-4 disodium phosphate (CA4DP; 250 mg/kg), ZD6126 (200 mg/kg) and 5,6-dimethylxanthenone-4-acetic acid (DMXAA, 20 mg/kg). Tumour oxygenation ($pO_2$) was estimated using the Eppendorf histograph. Single dose radiation (240 kV x-rays) was given locally to the tumour and response assessed using either growth delay (time for tumours to reach 3x treatment volume) or tumour control (percent of animals showing tumour control at 90 days).
**Results:** The mean ($\pm$ 1 SE) percent $pO_2$ values $\leq$ 5 mmHg measured in control tumours was 45 $\pm$ 5. At 1-hour after giving CA4DP or ZD6126 or 3-hours after DMXAA (times of maximum blood flow reductions) these were significantly (Student's t-test; p<0.05) increased to 91 $\pm$ 4, 73 $\pm$ 7 and 87 $\pm$ 9, respectively. Irradiating tumours and then injecting VDAs within 1-hour enhanced radiation response by factors of 1.2 (CA4DP), 1.1 (ZD6126) and 1.3 (DMXAA). Giving the radiation 1-hour after CA4DP or 3-hours after DMXAA resulted in a protective response, the enhancements being 0.96 and 0.74, respectively.
**Conclusions:** VDAs increased tumour hypoxia and irradiating at the time of this hypoxia induction reduced radiation response. Such an effect could impact the combination of VDAs and radiation when given in a clinically relevant fractionated schedule. Additional studies are now being performed to determine the time-dependency for the disappearance of this hypoxic resistance.
Supported by a grant from the Danish Cancer Society

### 6.4

### CYTOKINE MODULATION OF INOS EXPRESSION IN HUMAN TUMOUR CELLS ENHANCES RADIATION RESPONSE AND ACTIVATION OF BIOREDUCTIVE DRUGS

S Singh , EC Chinje , IJ Stratford
Ireland, The University of Manchester, Manchester

Hypoxia regulates the expression of cytokine-inducible nitric oxide synthase (iNOS) gene which catalyses the conversion of L-arginine to L-citrulline and nitric oxide (NO) in the presence of $O_2$ as a co-factor. iNOS can activate hypoxia selective bioreductive drugs and NO can sensitise hypoxic cells to radiation. Thus, in this study we determined the contribution of tumour iNOS to radiation and bioreductive drug sensitivity in air, hypoxia and at intermediate $O_2$ tensions.
Human breast tumour cells (MDA231) were exposed to cytokine-mix (CM) {IFN-γ (100ng/ml) + LPS (50μg/ml)} for 24h in air, anoxia or different $O_2$ tensions (0.02%-2%). CM was then washed out from cultures and cells re-incubated for further 24h in the different $O_2$ tensions, at which time cells were either exposed to the drugs (RSU1069 or TPZ) for 3h or irradiated at 37$^0$C and cell survival assessed by clonogenic assay. At the time of drug exposure, samples of cells were harvested and NADPH-dependent iNOS-reductase activity (NOSR), total iNOS activity (citrulline assay) and NO production (Griess assay) were determined.
NO output reached maximum levels 24hr after cytokine exposure at 2% $O_2$ (36μM compared to 1.62μM for untreated cells). This increased NO production had no impact on radiosensitivity of aerobic cells, but at lower $O_2$ range (≤1%$O_2$), a positive correlation between NO release and increase in radiation response was found. For example, at 1% $O_2$ where in uninduced cells the OER was 1.13; when cells were treated with CM the OER increased to 1.84. This increase in OER was abolished when the iNOS inhibitor L-NMMA was present during induction and irradiation of the cells. CM resulted in 2.16-fold increase in NOSR activity (7.23 compared to 3.34 nmol/mg protein for untreated cells). Cytotoxicity ratios for air/anoxia were 3.5 (untreated) and 8.25(CM) for TPZ, and 1.94 (untreated) and 3.43 (CM) for RSU1069, respectively. For air/0.5%$O_2$ ratios were 2.6 (untreated) and 6.0 (CM) for TPZ, and 1.26 (untreated) and 1.71(CM) for RSU1069, respectively.
This study shows that, at intermediate $O_2$, characteristic of hypoxic solid tumours, iNOS enzyme can catalyse activation of hypoxic cytotoxins TPZ and RSU1069. We also demonstrate that, NO produced endogenously can contribute to overall tumour radiosensitivity.

© 2004 Cancer Research UK

### 6.5

**EXPERIMENTAL PRECLINICAL THERAPY, USING [$^{131}$I]MIBG, OF TUMOURS TRANSFECTED WITH THE NORADRENALINE TRANSPORTER GENE**

M Boyd , SC Ross , J Owens , RJ Mairs
CRUK Beatson Laboratories, Glasgow, UK

Radiolabelled meta-iodobenzylguanidine (MIBG), an analogue of neuroadrenergic blockers, is used for imaging and treatment of neural crest-derived tumors, such as neuroblastoma and phaeochromocytoma, which express the targeted noradrenaline transporter (NAT). To determine whether [$^{131}$I]MIBG could also treat other tumor types, we transfected UVW human glioma cells with the NAT gene. These were used for the evaluation of radiopharmaceutical uptake *in vitro* and for the establishment of nude mouse xenografts.
By virtue of NAT gene transfer, the accumulation of [$^{131}$I]MIBG was enhanced 25-fold in UVW cells. The distribution of MIBG in tumour-bearing nude mice was compared by measurement of radioactivity in tissues excised from the animals. Twenty four hours after administration of [$^{131}$I]MIBG tumours not expressing the NAT took up only low levels of activity whereas the concentration of radiopharmaceutical in NAT bearing experimental tumours was 3.17 percent of injected dose per gram of tissue. In contrast, the concentration of MIBG was low in liver, kidney, lung thyroid and marrow.
I.p. injection of [$^{131}$I]MIBG significantly suppressed the growth of UVW/NAT tumours in a dose-dependent manner .Control tumours reached a ten-fold increase in volume in 17.2 ± 2.1 days, whereas the volume of tumours treated with the 14 MBq of [$^{131}$I]MIBG produced a cure rate of 100%. There was no evidence of thrombocytopenia and no significant difference in platelet count observed thirty days after treatment with a [$^{131}$I]MIBG . Likewise, the clonogenic capacity of pluripotential stem cells was not adversely effected by [$^{131}$I]MIBG treatment.
These results suggest that tumour-specific transfection of the NAT gene is a promising therapeutic strategy with minimal dose limiting organ toxicity.

### 6.6

**ENHANCEMENT OF TARGETED RADIOTEHRAPY IN NEUROBLASTOMA: A NOVEL GENE THERAPY APPROACH**

M Boyd , E Cosimo , AG McCluskey , AM Clark , T Robson , HO McCarthy , MR Zalutsky , RJ Maits
[1] CRUK Beatson Labs, Glasgow, United Kingdom, [2] Dept Radiology, Duke University, Durham, United States, [3] Centre for Biomolecular Sciences, Ulster University, coleraine, United Kingdom

Neuroblastoma, is one of the most common solid tumours in children. As the cure rate of neuroblastoma is low, new therapeutic approaches are necessary. The radiopharmaceutical [$^{131}$I]MIBG has produced encouraging results (long term remissions and palliation) in neuroblastoma patients and increased effectiveness could be obtained by increased concentration of the [$^{131}$I]MIBG in malignant cells. We previously reported that transfection of the NAT gene into neuroblastoma cells, induced the expression of a functional transporter which improved the active uptake of [$^{131}$I]MIBG and resulted in dose-dependent toxicity to the host cells.
A construct with radiation-inducible WAF1 promoter driving NAT should be able to upregulate the synthesis of the NAT in neuroblastoma cells. This strategy involves WAF1/NAT transfection followed by an initial dose of radiation in the form of [$^{131}$I]MIBG (concentrated preferentially by neuroblastoma cells), facilitating the tumour-specific overexpression of NAT. A second administration of [$^{131}$I]MIBG should be avidly concentrated by target tumour cells, leading to their sterilisation.
We transfected a neuroblastoma cell line (SK-N-BE) with a plasmid containing the GFP cDNA controlled by the radiation-inducible promoter of WAF1. According to FACS analysis, a 4 Gy dose of gamma radiation increased the GFP protein level to 11.7 times the unirradiated cells protein level. Using the same conditions, preliminary studies showed that low doses of targeted radionuclides of different radiation quality ($\alpha$–, $\beta$– and Auger emitters), [$^{123}$I]MIBG (0.5MBq/ml), [$^{131}$I]MIBG (0.5MBq/ml) and [$^{211}$At]MABG(50 nCi/ml) increased GFP protein level to at least 3.5 times the unirradiated cells protein level. These encouraging results suggest that the promoter of WAF1 is a promising means of driving the over-expression of the NAT gene in neuroblastoma cells in a radiation-dependent manner.

### 6.7

**RADIATION-INDUCED BYSTANDER SIGNALLING IN TARGETED GLIOMA CELLS**

C Shao , VC Stewart , M Folkard , BD Michael , KM Prise
Gray Cancer Institute, Northwood, United Kingdom

Bystander responses have been reported to be a major determinant of the response of cells to radiation exposure at low doses, including those of relevance to therapy. Human glioblastoma T98G cell nuclei were individually irradiated with an exact number of helium ions using a single-cell microbeam. It was found that when only one cell in a population of ~1200 cells was targeted, with one or five ions, cellular damage measured as induced micronuclei was increased by 20%. The level of bystander response was independent of whether radiation was targeted through the nucleus or cytoplasm only. However when c-PTIO, a nitric oxide-specific scavenger was present in the culture medium, the micronuclei yields reduced to the predicted values. By using DAF-FM, NO was detected *in situ*, and it was found that NO-induced fluorescence intensity in the irradiated population where 1% of cell nuclei were individually targeted with a single helium ion was increased relative to control with around 40% of the cells showing increased NO levels.
We have also detected bystander interactions between T98G glioma cells and normal primary human fibroblasts (AG01522), co-cultured within the same dish. A fraction of cells within one population was individually targeted through the nucleus with exactly one or five helium particles ($^{3}He^{2+}$) and micronuclei formation in the bystander nonirradiated population was measured. When only 1% of cell nuclei were irradiated, the NO level in the T98G population was increased by 31% and the ROS level in the AG0 population was increased by 18%. Treatment of cultures with c-PTIO abolished the bystander micronuclei induction in nonirradiated AG01522 cells but only partly attenuated the bystander response in nonirradiated T98G cells and this could be eliminated by treatment with DMSO. This suggests that differential mechanisms involving NO or ROS mediated signaling, dependent on the cell type targeted, were involved.

### 6.8

**RADIOTHERAPY FRACTIONATION: RESULTS OF A PILOT STUDY TO THE NCRI STANDARDISATION OF RADIOTHERAPY (START) TRIAL**

J Yarnold [1], A Ashton [2], J Bliss [3], B Broad [2], J Homewood [3], C Jackson [3], J Regan [1], J Haviland [3], S Bentzen [4], JR Owen [2]
[1] United Kingdom, Royal Marsden Hospital, Sutton, [2] United Kingdom, Cheltenham Oncology Centre, Cheltenham, [3] United Kingdom, Institute of Cancer Research, Sutton, [4] United Kingdom, Gray Cancer Institute, Northwood

**Aim:** To test the effects of fraction size > 2.0Gy on i) late normal tissue response and ii) tumour control in the breast after tumour excision and radiotherapy for early breast cancer.
**Methods:** 1410 women with T1-3 N0-1 M0 invasive breast cancer were randomised between 1986-98 into one of three radiotherapy regimens after local excision of early stage breast cancer; 50Gy in 25 fractions (F) vs two dose levels of a test schedule giving 39.0Gy or 42.9Gy in 13F over 5 weeks. Fractions sizes were 2.0Gy, 3.0Gy and 3.3Gy respectively. The primary endpoint was late change in breast appearance compared to post-surgical appearance scored from annual photographs scored blind to treatment allocation. Secondary endpoints included palpable breast induration and ipsilateral tumour recurrence.
**Results:** With a minimum follow up of 5 years, the risk of scoring any change in breast appearance after 50Gy/25F, 39Gy/13F and 42.9Gy/13F was 35.4% 27.4% and 42.3% respectively, from which an a/$\beta$ value of 3.6Gy (95% CI 1.8 - 5.4) can be derived. The $\alpha/\beta$ value for palpable breast induration was 3.1Gy (95% CI 1.8 - 4.4). Local tumour control at 10 years was 87.0%, 85.6% and 88.6% respectively, from which a/$\beta$ value of 4.1Gy (95% CI 1.0 – 9.7) can be derived.
**Discussion:** The estimated $\alpha/\beta$ value for tumour control is imprecise, but appears lower than that for squamous carcinomas and comparable to those of late reacting normal tissues. If the UK START trial confirms these findings, there will be a strong case for testing a regimen of whole breast radiotherapy based on 5 fractions of intensity modulated radiotherapy (proposed UK FAST trial)
**Conclusion:** the data are consistent with the hypothesis that fraction sizes > 2.0Gy are safe and effective when irradiating the breast. They strengthens the case for adjusting dose intensity by modulating fraction size rather than fraction number, an approach proposed for evaluation in the UK IMPORT trial.

© 2004 Cancer Research UK

